# Mucoadhesive polymers in substance-based medical devices: functional ingredients or what else?

**DOI:** 10.3389/fdsfr.2023.1227763

**Published:** 2023-08-04

**Authors:** Barbara Vigani, Silvia Rossi, Giuseppina Sandri, Maria Cristina Bonferoni, Carla M. Caramella

**Affiliations:** Department of Drug Sciences, University of Pavia, Pavia, Italy

**Keywords:** substance-based medical devices, mucoadhesion, mucoadhesive polymers, mechanisms, regulation

## Abstract

The paper is intended to deal with the regulatory status of the family of substance-based medical devices (SB-MD) which contain mucoadhesive polymers. Mucoadhesive formulations are mainly intended for oral/buccal, gastro-esophageal, nasal, or vaginal administration routes. They contain one or more substances/polymers of either natural, synthetic or semi-synthetic origin endowed with mucoadhesive properties. These are complex substances whose chemical-physical properties are in general well characterized. Hydration and water retaining properties, gel formation, lubricating properties are example of functional characteristics that may be involved in mucosal interaction. However, there are still uncertainties as to the underlaying mechanisms. The idea is to provide support, to the understanding of the prevailing mechanisms of action of the family of SB-MD that exploit mucoadhesion phenomenon to exert the intended therapeutic action. A case study on Hyaluronic acid as a typical representative of mucoadhesive polymers, is presented. The correct understanding of the mechanism of action of the substances/polymers involved in SB-MD is pivotal to a smooth and successful submission to the involved regulatory bodies to a positive assessment and to the final approval.

## 1 Introduction

The more general term bioadhesion commonly defines the adhesion of a substance (i.e., polymeric-based products, such as dosage forms or medical devices) to a biological tissue: the substance-biologic tissue adhesive interaction allows an intimate contact between the two materials for an extended period of time. The higher the strength of the adhesive interaction, the higher the residence time of the substance on the biologic tissue. When the biologic tissue is represented by the mucus layer that covers a mucosal tissue, the phenomenon is referred to as mucoadhesion (Edsman and Hägerström, 2005; Smart, 2005; Cook and Khutoryanskiy, 2015).

**FIGURE 1:**
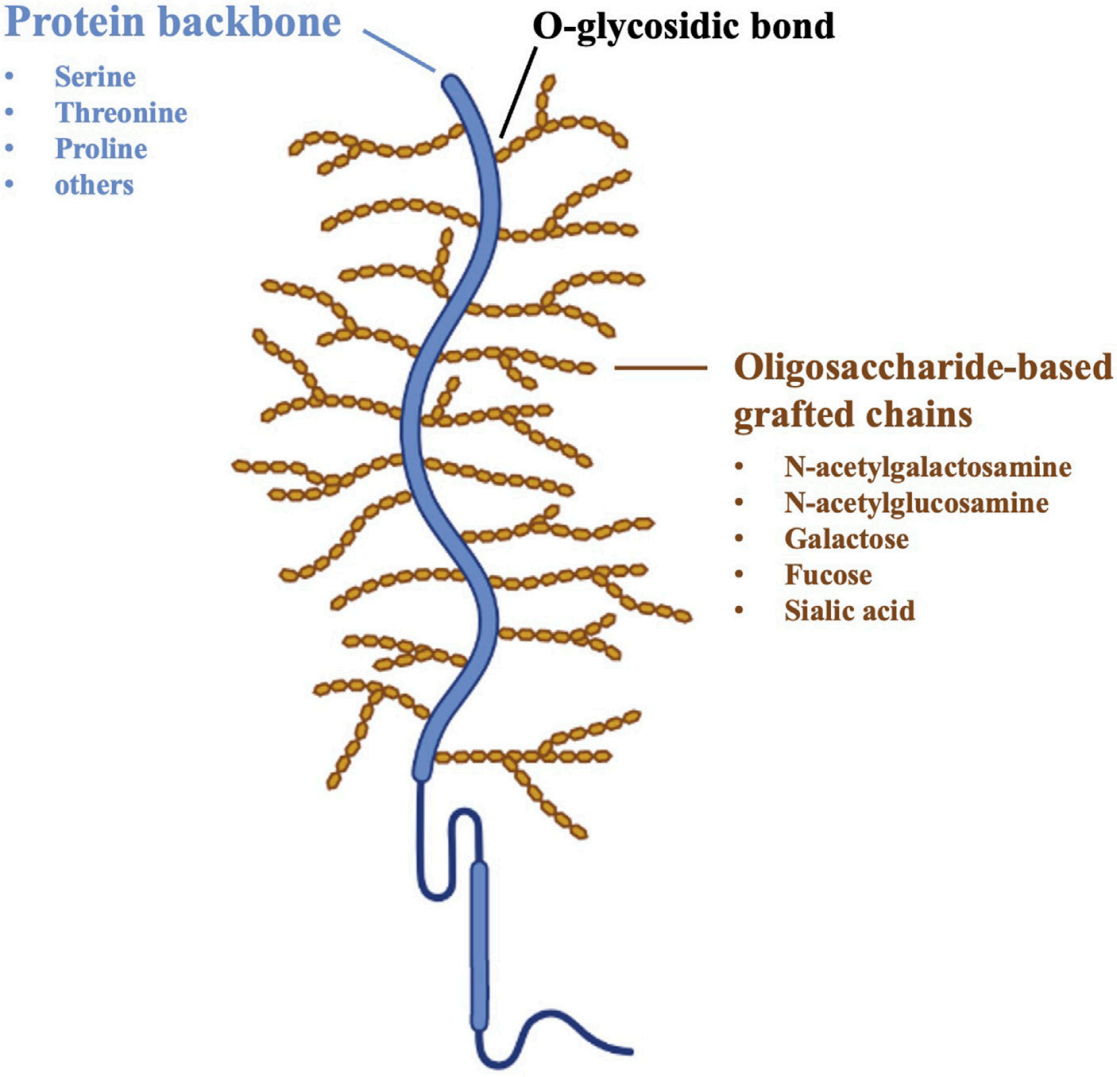
Schematic structure of mucin glycoproteins (Cook and Khutoryanskiy, 2015).

To provide a detailed understanding of the mucoadhesion phenomenon, a brief description of the structure of mucosal tissues, in particular mucus composition, is mandatory. The mucus layer consists of a highly hydrated viscoelastic gel network; it is composed by water (up to 95% by weight), glycoproteins (generally no more than 5% by weigh), inorganic salts, carbohydrates and lipids. Mucins are a family of soluble glycoproteins, which are responsible for the mucus gel-like structure due to their high molecular weight (MW) and ability to form complexes as a result of intermolecular disulfide bridges and hydrophobic interactions. Each mucin subunit consists of protein-based backbones (12%–17% of the total mucin weight), including 70% of serine, threonine and proline by weight, and oligosaccharide-based grafted chains, made of N-acetylgalactosamine, N-acetylglucosamine, galactose, fucose and N-acetylneuramic acid. More than 63% of the protein backbone is covered with oligosaccharide chains, with the remainder structure being non-glycosylated. Most mucins are characterized by a net negative charge due to the presence of carboxylate groups (sialic acid) and ester sulfates at the terminus of some sugar units; the pKa value of these acidic groups is approximately 1.0–2.6, resulting in their complete ionization under physiological conditions (Figure 1) (Smart, 2005; Cook and Khutoryanskiy, 2015).

Since the mid-eighties formulators have tried to exploit mucoadhesion phenomenon to ameliorate the performance of the dosage forms intended for mucosal application, basically through the oral/buccal, gastro-esophageal, nasal and vaginal administration routes (Kumar et al., 2020; Karavasili et al., 2021). Even though ocular and vesical epithelia are not classified as mucosal tissues, also the formulations intended to treat ocular or vesical diseases often claim muco/bioadhesion properties, due to the presence of mucin-like coverage. Mucoadhesive systems may belong to the category of medicinal products, but, most frequently, they are medical devices or even cosmetics; several of them have been well received and are currently available on the marketplace. The substances that play the major role in such systems are the so-called mucoadhesive polymers. A list of the most used polymers is reported in Table 1.

**TABLE 1:** Classification of mucoadhesive polymers based on generation, charge, solubility and molecular interactions (Bandi et al., 2021; Karavasili et al., 2021).

		Examples	References
Generation	I (traditional polymers)	Cationic and anionic polymers	Sosnik et al. (2014)
	II (functionalized polymers)	Thiolated polymers	Duggan et al. (2017), Leichner et al. (2019), Federer et al. (2021), Knoll et al. (2021), Summonte et al. (2021)
		Acrylated polymers	Agibayeva et al. (2020)
		Boronated polymers	Surendranath et al. (2022)
Charge	Cationic polymers	Chitosan	Sandri et al. (2014), Kumar et al. (2016), Ways et al. (2018), Collado-González et al. (2019), Freitas et al. (2020), Mura et al. (2022)
	Anionic polymers	PAA and its cross-linked polymers (carbomers), sodium alginate, carrageenan, gelatin, gums, sodium carboxymethylcellulose (NaCMC)	Dolci et al. (2020), Agibayeva et al. (2020), Göbel et al. (2021), da Silva et al. (2022), Yermak et al. (2022)
	Non-ionic polymers	Hydroxypropylmethylcellulose (HPMC), hydroxyethylcellulose (HEC) and methylcellulose (MC)	da Silva et al. (2022)
Solubility	Water-soluble polymers	Hydroxypropylcellulose (HPC), NaCMC	da Silva et al. (2022)
	Water-insoluble polymers	Ethylcellulose (EC), polycarbophil	da Silva et al. (2022)
Interaction	Electrostatic	Chitosan	Sandri et al. (2014), Kumar et al. (2016), Ways et al. (2018), Collado-González et al. (2019), Freitas et al. (2020), Mura et al. (2022)
	Covalent	Cyanoacrylate	Agibayeva et al. (2020)
	Hydrogen bond	Hyaluronic acid, poly(vinyl alcohol) (PVA), PAA and poly(hydroxyalkyl methacrylate)	Tamburic and Craig (1995), Pritchar et al. (1996), Leitner et al. (2003), Patel et al. (2003), Sandri et al. (2004), Fallacara et al. (2018), Estrellas et al. (2019), Vigani et al. (2019), Dovedytis et al. (2020), Dalei and Das (2022)

The present paper is not intended to be the hundredth review on mucoadhesive polymers, however, for the benefit of the reader, a list of references to published comprehensive review papers is provided hereafter (Roy et al., 2009; Cook and Khutoryanskiy, 2015; Yermak et al., 2022; da Silva et al., 2022; Rossi et al., 2014; Sandri et al., 2015; Caramella et al., 2015; Rossi eta l., 2018). In these reviews the various chemical categories of mucoadhesive polymers together with the relevant physical-chemical (MW, hydrophilicity, cross-links) and mechanical (cohesiveness) properties are discussed as well as their possible applications in the biomedical field. Furthermore, chemical functionalization of native polymers, such as thiolation (Duggan et al., 2017; Leichner et al., 2019; Federer et al., 2021; Knoll et al., 2021; Summonte et al., 2021), conjugation with boronate groups (Surendranath et al., 2022) and methacrylation (Agibayeva et al., 2020) has also been proposed to improve the mucoadhesive properties.

Even though the chemical-physical and mechanical properties are well characterized, mucoadhesive polymers are complex substances and there are still uncertainties as to their mechanism of action. The important question that needs to be answered in view of their possible employment in substance-based medical devices (SB-MD) is: are they simply functional excipients or do they play any unforeseen biological effect? Why is it not so simple to answer this question? It is necessary to go back to the mucoadhesion phenomenon and its complexity.

## 2 Mucoadhesion process and proposed theories

**FIGURE 2:**
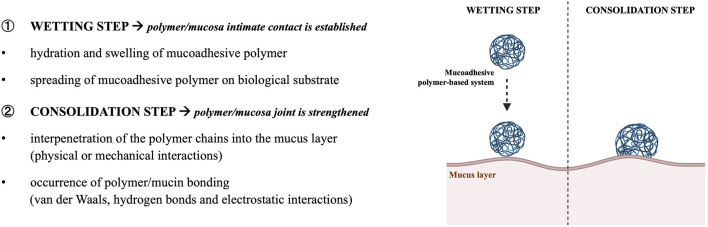
The two steps of mucoadhesion process.

As anticipated, the mucoadhesion process occurs at the interface between the adhesive substance and the superior layer of the mucosa. The first step of this process is the formation of an intimate contact (wetting step) between the substance and the mucus. In the pharmaceutical field, the nature of the dosage form/medical device may impact this process step. In the case of semisolids or liquids, the intimate contact with the mucosal tissue results from dosage form/medical device wetting and/or spreading, which are responsible for an increase in the contact area. Instead, as far as dry, and not fully hydrated dosage forms/medical devices, their wetting, hydration and swelling promote a more intimate contact with the mucosa. In a second step (consolidation step) of the mucoadhesion process, an interpenetration of the hydrated/swollen polymeric matrix (dosage form/medical device) and the mucus gel network occurs (Figure 2) (Edsman and Hägerström, 2005; Zahir-Jouzdani et al., 2018).

Indeed, both wetting and interpenetration are the results of more complex interaction between the formulation and the underlying tissular/cellular environment. Decades of research on this topic have allowed to develop six theories that explain the mechanism/s underlying the adhesion of a substance (i.e., polymer-based product) to mucosal surfaces. Here below brief description of each theory is provided (Reinhart and Peppas, 1984; Smart, 2005; Roy et al., 2009; Carvalho et al., 2010; Khutoryanskiy, 2011; Shaikh et al., 2011; Shinkar et al., 2012; Sosnik et al., 2014; Cook and Khutoryanskiy, 2015; de Lima et al., 2022).

### 2.1 Wetting theory

The wetting theory mainly applies to liquids or substances with low viscosity (hereby referred to as “adhesive candidate”), endowed with a high affinity to the mucus layer and, thus, a good capability to spontaneously spread onto the mucosa surface. The adhesive candidate-mucosa affinity can be evaluated through the contact angle method: the lower the contact angle, the higher the affinity between the substance and the mucosa. The contact angle indicates the degree of wetting when a liquid (i.e., the adhesive candidate) and a solid (i.e., the mucosa) interact: a contact angle equal or close to zero indicates an adequate spreadability of the adhesive candidate onto the mucosal tissue, that is a prerequisite for the mucoadhesion (Peppas and Sahlin, 1996; Mathiowitz et al., 1999; McBain and Hopkins, 2002; Shaikh et al., 2011).

### 2.2 Electronic theory

The electronic theory postulates that the adhesive candidate and the mucosal tissue are characterized by different electronic structures. Thus, after contact of adhering surfaces, an electron transfer occurs leading to the formation of a double electronic layer at the adhesive candidate-mucus interface; the result is an electrostatic attraction between the two surfaces that is responsible for mucoadhesion (Mathiowitz et al., 1999; Asati et al., 2019).

### 2.3 Adsorption theory

The adsorption theory predicts that mucoadhesion is achieved via specific interactions (primary and secondary bonds) between the adhesion candidate and the mucosal tissue. After contact of adhering surfaces, they mainly interact by hydrogen bonds and Van der Walls forces; hydrophobic interactions may play an important role especially when the adhesive substance (i.e., a polymer) has an amphiphilic nature. According to this theory, such interactions, although they are individually weak, are the main contributors to the mucoadhesion; a great number of interactions at the adhesive candidate-mucus interface can result in an intense adhesive phenomenon (Ahuja et al., 1997; Mathiowitz et al., 1999; Huang et al., 2000; Lee et al., 2000; Hägerström et al., 2003; Salamat-Miller et al., 2005).

### 2.4 Diffusion theory

The diffusion theory postulates that the polymeric chains of the adhesive candidate and the mucus glycoproteins (i.e., mucins) interpenetrate to a sufficient depth to create semi-permanent adhesive bonds. The interdiffusion phenomenon mainly depends on the diffusion coefficient and the time of contact between the adhesive candidate and the mucus layer; it is generally enhanced when polymeric chains and mucins have similar chemical structures and are mutually soluble. The existence of concentration gradients is the driven force that promotes the diffusion of the polymeric chains within mucus network and, in turn, the mucin chains into the adhesive polymeric matrix until an equilibrium interpenetration depth is achieved. According to the literature, the interpenetration degree of the polymeric chains is also affected by certain properties of the polymer, such as MW, flexibility, hydrophilicity and cross-linking density (Peppas and Buri, 1985; Duchene et al., 1988; Jimenez-Castellanos et al., 1993).

### 2.5 Mechanical theory

The mechanical theory describes the effect of the surface roughness of the mucosal tissue on the adhesion of liquids due to their interlocking with the mucosa itself; more in detail, such systems fill the irregularities of the rough surface, which are responsible for an increase in the interfacial area available for adhesive interactions (Lee et al., 2000).

### 2.6 Fracture theory

The fracture theory relates the strength of the adhesive bonds to the forces required to detach the two adhering surfaces after contact. This theory is different to the others: the mucoadhesion phenomenon is described by the fracture (or detachment) strength at the interface and is not related to the factors (i.e., polymer chemical structure, molecular weight, hydrophilicity, chain flexibility) that could affect the formation of adhesive bonds between the adhesive candidate (i.e., solid system) and the mucus layer. Since such a theory is based on mechanical considerations, it is also necessary to consider the other theories to better understand the mucoadhesion phenomenon in its entirety (Gu et al., 1988; Asati et al., 2019).

**FIGURE 3:**
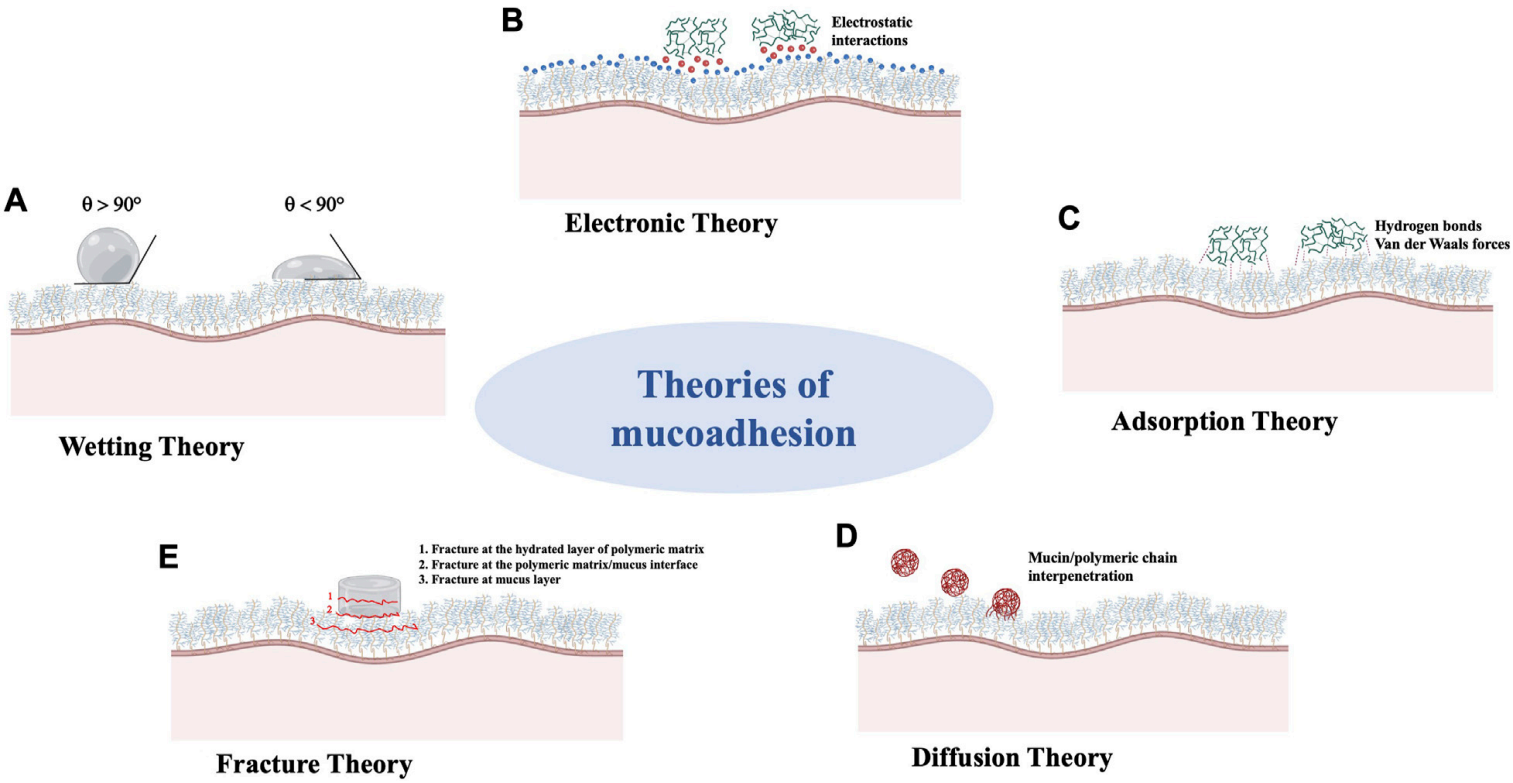
Schematic representation of theories which explain the mechanism/s underlying the mucoadhesion phenomenon. **(A)**: Wetting Theory; **(B)**: Electronic Theory; **(C)**: Adsorption Theory; **(D)**: Diffusion Theory; **(E)**: Fracture Theory.

A pictural representation of the different theories is provided in Figure 3. None of these theories fully explain mucoadhesion, but several of these theories can be combined to obtain a comprehensive picture of the phenomenon. Depending on the nature of the system (i.e., liquid or solid), some theories are more applicable than others, but the relevance of the various theories is also strictly related to mucus layer. The high variability in mucus properties, such as viscoelasticity, thickness (from 50 to 450 μm in the stomach to less than 1 μm in the oral cavity), pH and sensitivity to various external stimuli, depends on both the location in the organism (ocular, nasal, buccal, respiratory, gastric, intestinal, cervico/vaginal mucus layers) and the physio-pathological conditions (i.e., over-production of mucus in SARS-CoV-2 infections) and it must be taken into account throughout the various development phases of mucoadhesive materials (Bayer, 2022).

## 3 The regulatory issue

Most importantly, all these theories were postulated between the late eighties and the first decade of the twenties, wherein the need to identify the prevailing mucoadhesion mechanism of a given substance were not so stringent. The need for such a distinction has become evident with the advent of the new European Regulation for medical devices 2017/745 (MDR) (hereafter referred as New Regulation) eventually implemented in May 2021, accordingly which a distinction between medicinals and SB-MD (so-called borderline products) became mandatory suggesting that the discriminant between the two should be the mechanism of action of the so-called active ingredients. Mucoadhesive systems are an example of borderline product, presently available on the market as both medicinals and medical devices, so they represent an interesting case-study from the regulatory point of view.

The aim of this section is to provide a rationale thinking as well as bibliographic data to support the prevailing mechanisms of action (principal mode of action) of SB-MD containing mucoadhesive polymers independently of the type of substance/s employed and the intended site of application. It is also implicit that if the mucoadhesive products contain other substances than the mucoadhesive polymers (for instance herbal extracts or similar), these should deserve specific consideration in view of the specific aim of demonstrating their mechanisms of action.

### 3.1 Definitions and historical highlights

SB-MD, like mucoadhesive products, belong to the broad family of medical devices that have been on the market for a long time under the scope of the previous legislation (European Union, 1993). They fall under the definition of medical devices as already present in Directive 93/42/EEC and adopted by the new Regulation, in art. 2, c. 1: “*Medical device means any instrument, apparatus, appliance, software, implant, reagent, material or other article intended by the manufacturer to be used, alone or in combination, for human beings for prevention, treatment or alleviation of disease, or of an injury, and which does not achieve its principal intended action by pharmacological, immunological or metabolic means (abbreviated Ph.I.M), in or on the human body*.*”*

According to the expert opinion expressed by Racchi et al. (2016), the term pharmacological means underlies all the Ph.I.M. mechanisms of action since immunological and metabolic modes of action are specific pharmacological actions. Presently the regulatory document uses the term pharmacological means (Ph. means) to indicate Ph.I.M means. Since the above definition of MD was released, it was clear that the definition of SB-MD overlaps in part with the definition of medicinal products. For this reason, since then they had been watched by MEDDEV (MEDical DEVices) Document (MEDDEV 2.1/3 rev. 3), being considered as borderline with medicinal products. According to MEDDEV guideline, “*The borderline nature stems from the fact that both have the same presentation (dosage form) and composition (one or more substances). Medical devices may also be intended to treat and prevent disease, along with other specific medical purposes*. *Therefore, the decisive criterion for the demarcation between the two categories is the principal mode of action of the product.,*” (MEDDEV 2.1/3 rev.3). In other words, for medical devices, it is necessary to demonstrate that they do not achieve their purpose by any pharmacological means.

Therefore, it is interesting to cite again the article by Racchi et al. since it contains interesting hints that might be useful when discussing the mechanisms of action of the mucoadhesive products thus defined. In the present article, the authors want to emphasize that the ‘mechanism of action’ and the ‘therapeutic effect’ of a product are different concepts. The same therapeutic effect might be achieved, in some cases through a pharmacological mode of action in other cases through a not pharmacological one. To quote an example relevant to the mucoadhesive materials (that is applicable to buccal, oropharyngeal, nasal, vaginal, etc.), the same effect (i.e., anti-inflammatory) may be reached by both a pharmacological means (inhibition of cyclooxygenase (NSAIDS)) and a physical mode of action (formation of a protective barrier to limit contact between tissue and external or internal irritating agents) (Racchi et al., 2016).

### 3.2 Invasiveness and the level of risk

SB-MD are generally considered invasive devices according to Art. 2, c.1 of the New Regulation: “*invasive device means any device which, in whole or in part, penetrates inside the body, either through a body orifice or through the surface of the body*”, which is the case of mucoadhesive products. The New Regulation (Art. 51) as well as the old regulation requires to appropriately classify the medical device based on the intended purpose of the devices and their inherent risks in accordance with Annex VIII. Such a classification, in the case of an invasive medical device should be based on appropriate consideration on the level site and duration of invasiveness, but especially on the type of functioning, hereafter implicitly referring the mechanisms involved in the interactions with the specific part of the body.

The rule 21 of the new/old Regulations defines the level of risk associated to SB-MD as follows: “*Devices that are composed of substances or of combinations of substances that are intended to be introduced into the human body via a body orifice or applied to the skin and that are absorbed by or locally dispersed in the human body are classified as: class III if they, or their products of metabolism, are systemically absorbed by the human body in order to achieve the intended purpose;—class III if they achieve their intended purpose in the stomach or lower gastrointestinal tract and they, or their products of metabolism, are systemically absorbed by the human body;—class IIa if they are applied to the skin or if they are applied in the nasal or oral cavity as far as the pharynx, and achieve their intended purpose on those cavities; and—class IIb in all other cases*”. If we should consider this rule, we could superficially conclude that, since the mucoadhesive materials are typically high molecular weight macromolecules and as such they are not systemically absorbed, the mucoadhesion products, independently on the site of application, should fall into the risk categories IIa or IIb, without prejudice to being considered as medical devices to all effects. It is reminded that what does make the difference between the class IIa and IIb is the level of clinical evaluation needed in support according to Chapter VI of the New Regulation.

### 3.3 Novelty introduced by the new regulation

In view of the above, it is necessary to consult the New Regulation (European Regulation for medical devices 2017/745 (MDR)), in particular the premise 59 that reads: “*Rules under the old regime applied to invasive devices do not sufficiently take account of the level of invasiveness and potential toxicity of certain devices which are introduced into the human body. To obtain a suitable risk-based classification of devices that are composed of substances or of combinations of substances that are absorbed by or locally dispersed in the human body, it is necessary to introduce specific classification rules for such devices. The classification rules should consider the place where the device performs its action in or on the human body, where it is introduced or applied, and whether a systemic absorption of the substances of which the device is composed, or of the products of metabolism in the human body of those substances occurs*”.

This statement was alerting but not helpful pending an updated definition of Pharmacological means. Indeed, independently of whether a substance or any metabolite are systemically absorbed or not, it is required to elaborate further on their mechanism of action, not to risk falling into the category of medicinal products. The New Regulation in Art. 4 (European Union, 2017) delegates the definition of Pharmacological means to the new panel of experts of the Medical Device Coordination Group (MDGD) established under Art. 103.

Eventually, in April 2022, the new MDCG 2022—5 “Guidance on borderline between medical devices and medicinal products under Regulation (EU) 2017/745 on medical devices” was published. This guideline has further elaborated the concept of pharmacological, immunological, or metabolic means as follows “*Pharmacological means is understood as an interaction typically at a molecular level between a substance or its metabolites and a constituent of the human body which results in initiation, enhancement, reduction or blockade of physiological functions or pathological processes*.” Examples of constituents of the human body (cell membranes, intracellular structures, RNA, DNA, proteins, i.e., membrane proteins, enzymes) as well as components of extracellular matrix, components of blood and components of body fluids. Examples of immunological means and metabolic means are also given in non-exhaustive lists. Based on the above, it is not yet understood whether the new rules will help experts in correctly elaborating the principal mode of action of their intended devices, and how the implications of such new definitions will be received by the Notified Bodies. By the way, it seems that the intended simplification tends to make the definition of the mode of action more complicated.

## 4 The case of mucoadhesive substances/polymers

The mucoadhesion theories, previously described in detail, have been functional to the characterization of mucoadhesive substances, either of natural, synthetic or semisynthetic polymers/materials, and still represent a useful tool in the development of new mucoadhesive materials and products. Besides that, the above theoretical classification provides a useful guide to correctly categorize the mechanisms involved within the frame of the physical means/mechanisms that are admitted for a substance used in a medical device.

Among the mucoadhesion theories previously described, the most accredited mechanisms are those predicated by wetting, adsorption and diffusion. All the three theories are based on week interactions between the mucosal surface and the system (i.e., hydrogen bonds, van der Vaals forces, etc.), but also on electrostatic forces and hydrophobic bonds; all are reversible in nature and non-specific. Even when covalent bonds are involved, like in the case of thiomers, these are created with mucin and not directed to any cellular constituents. All these bonds contribute to strengthen the mucoadhesive joint.

At this point it is useful to recall the regulatory definition of physical means, in opposition to pharmacological means, as predicated by the MDCG document April 2022. Authors’ comments are bracketed: “*According to the above document, medical device’s principal intended action (that is the effect) is achieved by physical means (that is the mechanism/mode of action) including mechanical action, physical barrier such as a film, lubrication, heat transfer, radiation, ultrasound, replacement of or support to organs or body function*)*. Furthermore, hydration or dehydration and pH modification may also be means by which a medical device achieves its principal intended action*.”

In the following section, the examples of hyaluronic acid (HA) and polyacrylic acid (PAA) are reported to elucidate how the interactions mentioned above, on which the mucoadhesion mechanisms are based, can be mechanistically explained, and how they have been experimentally demonstrated at the mucoadhesive joint.

## 5 Case-study 1: the example of hyaluronic acid

HA is a highly prevalent mucopolysaccharide present in human fluids and tissues that plays an important role in mucociliary clearance, tissue hydration and the defense against the spread of micro-organisms and toxic substances. It is used in many fields of medicine, for example, ocular surgical procedures for the relief of dry eyes, intra-articular injection in the treatment of osteoarthritis and wound care as well as in a number of mucoadhesive products (mouth washes, nasal sprays, aerosols, oropharyngeal preparations, antiacid preparations, vaginal moisturizing gels, but also anti-reflux medications, rectal and colon washes) (Fallacara et al., 2018).

HA is chosen as a typical representative of mucoadhesive substances. This choice is emblematic, since, in a certain way, HA represents the worst case in the scenario of mucoadhesive polymers. Indeed, HA has been considered a borderline substance which might also have specific interactions with cellular components when administered through routes other than the above-described mucosal sites (Vasvavi et al., 2020). This peculiar aspect of HA is beyond the scope of the present paper that aims solely at explaining the mucoadhesive properties of HA and clarifying whether the relevant claimed mechanisms can be explained in terms of chemical, physical or mechanical means. HA’s claimed mode of action is hydration and related phenomena, such as swelling, lubricant and plasticizing effects, bio adhesion and bonding to mucosal components and protective effect. In the following paragraphs it is explained how hydration helps swelling, lubrication and ultimately bio adhesion (Vigani et al., 2019; Dovedytis et al., 2020).

### 5.1 HA interactions with mucus layer

#### 5.1.1 Hydration

**FIGURE 4:**
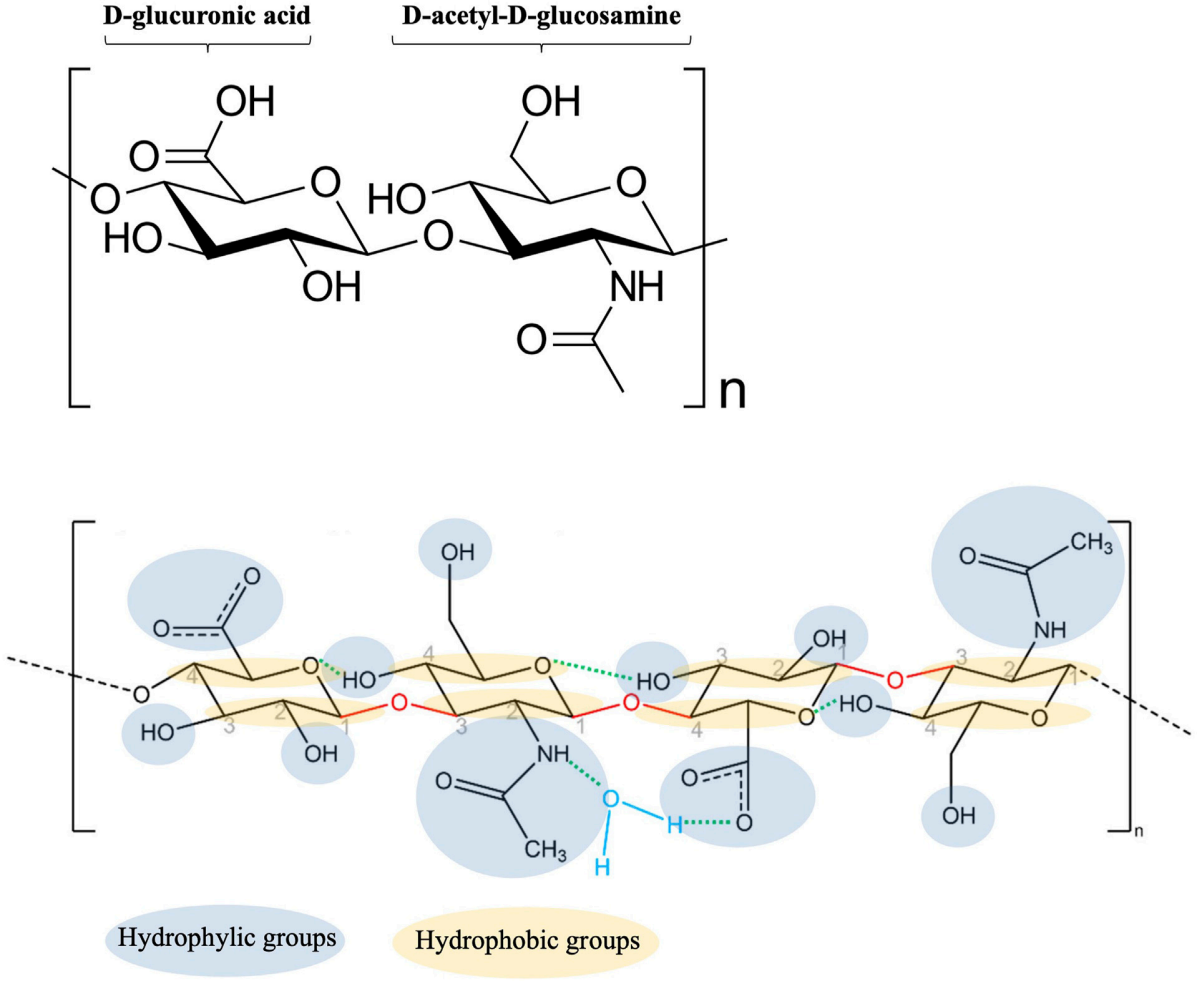
Schematic representation of HA-water bonding (modified from Dovedytis et al., 2020).

Since its carboxyl groups (COO^−^) are completely ionized at physiological pH, HA interacts with water molecules through hydrogen bonds. The negative charge can be balanced with mobile cationic ions (i.e., Na^+^, K^+^, Ca^2+^ and Mg^2+^), which are present in biological aqueous fluids. Therefore, once in contact with the mucosa, HA is persistently negatively charged and form salts with cationic ions (Fallacara et al., 2018). As shown in Figure 4, the positive charge of water dipole is attracted to the negatively charged carboxylate group of the glucuronic acid, while the negatively charged oxygen in water is attracted to the positively charged acetamido group of the N-acetyl-D-glucosamine. The unique water-retaining properties of HA explain its moisturizing effect (Vigani et al., 2019; Dovedytis et al., 2020).

The abundance of hydrophilic functional groups (-COOH), completely ionized at physiological pH, allows the formation of hydrogen bonds with the mucous membrane (Pritchard et al., 1996; Sandri et al., 2004). Therefore, hydration is fundamental to trigger the proper wetting of the mucosal substrate and the spreading of the formulation on the mucosa.

#### 5.1.2 Swelling

Besides that, the water-retaining properties of HA allow the polymer to swell, forming a viscous gel, and spread on the mucosa, maximizing the interaction between the polymer chains and the mucous network. In particular, the formation of hydrogen bonds increases the flexibility of HA chains, which become free to move within the mucus network, creating physical entanglements with mucins. Hydrogen bonding as well as the physical interpenetration between swollen HA chains and mucus network results in a mucoadhesive effect (Gu et al., 1988; Chatterjee et al., 2017). It is also important to note that the interaction between HA and mucosa is aspecific being mainly related to physico-chemical events such as polymeric chain interaction with water molecules, swelling and disentanglement, interpenetration through the mucous network and formation of new physical entanglements with mucins. Nobody would question that the one described above is a physico-chemical mechanism. It has also to be remarked that excessive hydration and swelling should be avoided since it will ease the removal of the mucoadhesive layer from the mucosa. However, this point has to do with the strategies to improve mucoadhesion by chemico-physical modifications of the polymer, not being related to the type of mechanism.

#### 5.1.3 Lubrication

Due to its water-retaining properties, HA acts as a lubricant; once in contact with the mucous layer, HA interacts with water molecules through hydrogen bonds and produces viscous gels exhibiting the so-called plasticizing effect (change of the material state/behavior from rigid to flexible) (Marcilla and Beltran, 2004), it can be concluded that, after hydration, HA swells and plasticizes the system, reducing the intermolecular frictions between the chains of the other polymeric components, while improving their motility and flowability. Nobody would question that this is a mere physical mechanism.

### 5.2 Combination with other polymers

HA if often combined with other polymers either natural, such as guar gum, or semi-synthetic ones like hydroxyethyl cellulose or synthetic ones, like carbomers or poloxamers. This combination allows for the formation of additional intermolecular interactions which strengthen the three-dimensional network. Interesting examples of combinations are given in the literature (Jiménez et al., 2007; Mayol et al., 2008). Especially polysaccharides that exhibit a water-binding capacity due to their high content in hydroxyl groups, should provide a synergistic effect leading to a moisturized and appropriately wetted mucosa and acting as a protective barrier for the entry of exogenous noxious agents (Hamza et al., 2008).

### 5.3 Considerations on HA mucoadhesive properties

This section will summarize the main conclusions about the principal mode of action of HA as used in mucoadhesive products. The cited cascade of effects (hydration, swelling, lubrication, that in turn trigger phenomena such as wetting of the mucosal surface, spreading of the formulation, interpenetration of the polymeric and mucin chains, interlocking due to weak and reversible bonds, strengthening of the mucosal interface, rheological synergism between polymers) all together exhaustively describe and explain the mucoadhesion phenomenon. Nobody would question that these are mere physical mechanisms. The result of the formation of this mucoadhesive interface is the prolongation of the residence time of the formulation on the target mucosa promoting the desired effect, i.e., optimal hydration of the tissue, protection against the insult of the acidic environment or any other noxious event. According to MDCG document (April 2022), the *physical barrier such as a film* is one of the mechanisms recognized by the legislator as a *physical mean.*

## 6 Case study 2: the example of polyacrylic acid and its derivatives

Polyacrylates are known to show adhesive behavior and acrylic-based polymers are widely used in biomedical and pharmaceutical applications depending on their molecular weight, functionalization and cross-linking (Leitner et al., 2003; Lam et al., 2021). The ones endowed with mucoadhesive properties are polyacrylic acid (PAA) and its derivatives. PAA was first described by Park and Robinson, (1987) as a mucoadhesive polymer capable of controlling the retention of a dosage forms in the stomach and even elsewhere in the gastro-intestinal tract.

Since the early years, PAA derivatives, such as carbopol (CP) and polycarbophil (PCP), have been proposed and extensively studied for the preparation of pharmaceutical and para-pharmaceutical formulations intended for both mucosal application and other administration routes (Tamburic and Craig, 1995; Carvalho et al., 2013). As mucoadhesive polymers they are basically intended to prolong the residence time of the dosage form at the application site; the high mucoadhesive potential of PAA derivatives is mainly exploited in vaginal drug delivery and to a lesser extent for in buccal, nasal and oral one. Moreover, due the versatility of their physico-technological properties, PAA derivatives are successfully used in the formulation of various semisolid and solid dosage forms, such as gels, pessaries, suppositories, but also granulates, tablets, micro- and nanoparticles (Wahlgren et al., 2009; Carvalho et al., 2013; Caramella et al., 2015a; Hanafi et al., 2019; Gosecka and Gosecki, 2021).

### 6.1 Hydration and swelling properties

In the case of PAA, hydration and swelling properties are strictly related. As in the case of HA, PAA and its derivatives are anionic polymers with hydrogel-forming moieties (carboxylic and hydroxyl groups), but contrary to HA, their polymeric backbones are more hydrophobic, meaning that PAA hydration and swelling properties are pH-dependent.

PAA is poorly soluble in water at low pH, causing structure shrinkage. It shows swelling properties under certain ionic strengths and salt concentrations in alkaline solutions. This is due to certain functional groups (-COOH) which are undissociated at acidic pH (less than 5) and dissociated at pH higher than 5.

In the early studies by Park and Robinson, (1987), it was envisaged that the mucoadhesive properties of PAA derivatives are due to the formation of hydrogen bonding between the carboxylic groups of PAA in the protonated (undissociated) form and the many hydrogen bonding sites of mucin macromolecules, which occurs mainly at acidic pH.

Thereafter, it has been confirmed (Patel et al., 2003) that, at pH < 4.0, the majority of the carboxylic groups are available in protonated form for the formation of conventional head-to-head H-bond dimers, whereas, at pH > 4, the majority of the carboxylic groups of both poly(acrylicacid) and the mucus glycoprotein are ionized.

On the contrary, the entanglement and the interpenetration of the bioadhesive polymer chains with the glycoprotein network are necessary for the formation of a mucoadhesive joint. These mechanisms require a tailored degree of swelling of the formed gel, which occurs at higher pH levels, thus explaining why polymer chain mobility and entanglements, and the consequent molecular interlocking, are favored in neutral/alkaline conditions.

A compromise between the number of available protonated carboxylic groups and the swelling properties was obtained by creating a series of cross-linked PAA derivatives. As it is often the case with polymers, the cross-linking, either chemically or physically induced, by reducing chain mobility, is functional to the modulation of the swelling properties.

### 6.2 PAA derivatives

PAA cross-linked derivatives, such as CP and PCP, are most commonly used as mucoadhesives. They show a high molecular mass and a high density of carboxylic acid groups. Therefore, at acidic pH, these polymers show minor swelling propensity due to a low percentage of dissociated acidic moieties. On increase in pH, additional charges result in electrostatic repulsion as well as osmotic forces within the polymeric backbone and uncoiling/expansion of the molecules lead to the desired degree of swelling of the polymer network (Zahir-Jouzdani et al., 2021). At the same time the cross-links prevent from an excessive swelling which would be detrimental to mucoadhesion.

Following the initial studies on PAA, the mechanisms of action of its derivatives have been thoroughly investigated and all the authors have come to the conclusion that hydrogen bonding significantly contributes to the mucoadhesion phenomenon, whereas swelling of the gel formed serves to consolidate the interlocking between polymeric chains (Shaikh et al., 2011). In an interesting paper (Patel et al., 2003), the authors have used various spectroscopic techniques (IR), nuclear magnetic resonance (NMR), and X-ray photoelectron spectroscopy (XPS), and differential scanning calorimetry (DSC) to investigate at the molecular level the interactions between mucin and Carbopol 934P. The formation of hydrogen bonds between mucus and the mucoadhesive agent (CP) has been shown by the displacement of IR absorption bands and by NMR resonances. In addition, differences in surface atom concentrations, between mixtures and separate components, were consistent with H-bonding between the components.

### 6.3 Combination/interaction with other polymers

CPs and PCPs are used in combination with other polymers such as hyaluronic acid in several medical devices (gels/suppositories) used for the treatment of vaginal dryness as shown in Table 2.

**TABLE 2:** Examples of mucoadhesive products registered as medical devices and available on the market are given in the Table 2. Commercial name, company, intended use are mentioned.

Device name	Manufacturer	Indication
ESOXX	APHARM S.R.L.	GERD
Gastrosoma REFLUX	LABOMAR S.R.L.	GERD
NEOBIANACID	ABOCA S.P.A. SOCIETA’ AGRICOLA	GERD
GENGIGEL	RICERFARMA SRL	Periodontal disease and other gingival tissue trauma
MUGARD	NORGINE B.V.	Oral mucositis
Episil	Camurus AB	Oral mucositis
Gelclair	Biokosmes S.R.L.	Oral mucositis
SAGINIL GEL	EPITECH GROUP S.R.L.	Vulvovaginitis/vaginal dryness secondary to atrophy
Gynexelle Hyalo-duo	ICIM INTERNATIONAL S.R.L.	Vulvovaginitis/vaginal dryness secondary to atrophy
IMMUNOVAG	DEPOFARMA S.P.A.	Vulvovaginitis/vaginal dryness secondary to atrophy

Besides that, the interaction between PAA derivatives and Poloxamers has been used to develop *in situ* gelling formulations, taking advantages of their pH dependent swelling properties (Zahir-Jouzdani et al., 2018). Other PAA derivatives, such as pegylated and thiolate polyacrylates have been proposed for optimizing the mucoadhesive properties (Leitner et al., 2003; Bayer et al., 2022).

### 6.4 Considerations on PAA derivative mucoadhesive properties

To conclude about the principal mode of action of PAA derivatives, as mentioned above, all authors concur to the conclusion that hydrogen bonding formation significantly contributes to mucoadhesion, whereas swelling of the gel formed serves to consolidate the interlocking between polymeric chains. At the same time, the peculiar pH dependent solubility of these polymers makes them suitable to formulate both solid and semisolid dosage forms. For similar solubility reasons the lubrication mechanism cannot be claimed for these materials. Nobody would question that the quoted mechanisms are mere physical mechanisms according to MDCG document (April 2022).

As a final consideration, both pharmaceutical product/medical devices are already present on the market whereby PAA derivatives are used as functional excipients with no prejudice with respect to their merely physical mechanism of action.

## 7 Examples of registered products

Examples of mucoadhesive products registered as medical devices and available on the market are given in the Table 2. A couple of products are briefly commented here below as an example.

### 7.1 Mucoadhesive gel for the treatment of mucositis: Gelclair

The gel used as a mouthwash helps the management of painful symptoms deriving from oropharingeal mucositis. Due to its mucoadhesive properties, it forms a protective film thus alleviating relief against pain caused by many insults, including chemio- and radiotherapy.

The composition of Gelclair (Caramella et al., 2015a) comprises, besides the vehicle, flavouring agents and preservatives, and the following mucoadhesive substances: PVP, sodium hyaluronate, maltodextrin. PVP and sodium hyaluronate are certainly the prevailing ones as responsible for the mechanisms of bioadhesion and barrier film formation, as pointed out in the following papers (Buchsel, 2008; Vokurka et al., 2011). The increased residence time of the formulation was also proved by means of a washability test using fluorescence markers (Rossi et al., 1999; Sandri et al., 2007; Caramella et al., 2015b).

The mode of action of mucoadhesive polymers, specifically hyaluronate sodium and PVP, is in line with the concept of physical means as required for a medical device. Besides that, it is expected that the level of risk assigned to Gelclair, based on rule 21, and related the level of injury of the underlying mucosa, could be higher in comparison to other mouthwashes.

### 7.2 Mucoadhesive gel for gastro-esophageal reflux syndrome: ESOXX

The product is a combination of hyaluronic acid, chondroitin sulfate, PVP, poloxamer 407, and contains xylitol C, sodium benzoate, potassium sorbate as preservative, flavoring agents and purified water. The gel acts as a physical barrier, protecting the gastro-esophageal mucosa against the attach of gastric juice (Di Simone et al., 2012). As for the composition, it is noted that PVP is considered an adhesive substance in general as suggested by its chemical nature, whereas chondroitin sulfate mainly favors the reparative processes of the underlying mucosa. Instead, the combination of hyaluronic acid with poloxamer represents a rather innovative solution since a synergic effect has been demonstrated between the two polymer types in promoting jellification and gel strengthening. Mayol and colleagues (2008) have demonstrated that the mechanical and mucoadhesive properties of poloxamer blends (F127 and F68), known to possess thermo-gelling properties, could be influenced by the addition of low molecular weight (150 kDa) HA. The authors evidenced that the presence of HA did not hinder the self-assembling process of poloxamers into micelles and, thus, did not significantly affect the thermo-gelling properties of poloxamer blends. On the counterpart, the addition of HA was responsible for an increase in poloxamer gel strength; the authors supposed that HA formed secondary bonds, in particular hydrogen ones, with micelles during poloxamer gelation, reinforcing the gel structure. Such a hypothesis was confirmed by PCS (Photon Correlation Spectroscopy) analysis: aggregates with hydrodynamic diameters higher than those of poloxamer micelles were measured when HA was added to poloxamer blends. Such results demonstrated that HA, in the hydrated state, allow micelles formation, movement and packing and interact with micelles through hydrogen bonds, improving the mechanical properties of the resulting poloxamer gels (gel network strengthening). In addition, a rheological synergism between poloxamer/HA gels and mucin dispersion was observed proving the mucoadhesive potential of the gels.

To note: the regulatory status of the medical devices registered in Italy can be found on the website of the Ministry of Health using link cited above. It is worth mentioning that presently the majority of products are still registered according to the old Directive, pending their re-evaluation according to the new Regulation.

## 8 Conclusion

The need to define the regulatory status of the family of substance-based medical devices (SB-MD) has become rather urgent in view of the complete implementation of the new Regulation (EU) 2017/745 which requires a clear demarcation between SD-MDs and medicinals. Mucoadhesive formulations are intended for oral/buccal, nasal, vaginal administration but even ocular and gastro-intestinal administration routes, therefore they do present different levels of invasiveness and associated risks. Independently of the route, the substances/polymers used are generally the same even though different in types and grades. The number of papers published on these substances and their pharmaceutical use has increased almost exponentially since the mid-eighties and witnesses the interest of scientists, engaged in pharmaceutical development both in academy and industry, for these ingredients. Much is known about their chemical-physical and pharmaco-technical properties. Also *in vitro* cytotoxicity as well as *ex vivo* and *in vivo* animal toxicology have been thoroughly investigated. Further to that, due to the high molecular weight, they are not absorbed through the common routes of administration, which simplify life in supporting their ADME pattern and toxicokinetic evaluation. In the literature information are also available on specific interactions of these substances with cellular components, which could evocate a pharmacological means, specifically when the substance is administered through parenteral routes, which is not typically the case of mucosal administration. Therefore, an attentive literature search should be sufficient to justify their use as functional excipients. The example of hyaluronic acid aims at indicating possible argumentations that could be used to support the prevailing mode of action of any specific mucoadhesive polymers and justify their use in medical devices. Same argumentations could also help in answering to request for clarification issued by the competent authorities European Commission, (2009), Suharyani et al., 2021.

## References

[B1] Agibayeva L. E. Kaldybekov D. B. Porfiryeva N. N. Garipova V. R. Mangazbayeva R. A. Moustafine R. I. (2020). Gellan gum and its methacrylated derivatives as *in situ* gelling mucoadhesive formulations of pilocarpine: *In vitro* and *in vivo* studies. Int. J. Pharm. 577, 119093. 10.1016/j.ijpharm.2020.119093 32004682

[B2] Ahuja A. Khar R. K. Ali J. (1997). Mucoadhesive drug delivery systems. Drug Dev. Ind. Pharm. 23, 489–515. 10.3109/03639049709148498

[B3] Asati S. Jain S. Choubey A. (2019). Bioadhesive or mucoadhesive drug delivery system: A potential alternative to conventional therapy. JDDT 9, 858–867. 10.22270/jddt.v9i4-A.3708

[B4] Bandi S. P. Bhatnagar S. Venuganti V. V. K. (2021). Advanced materials for drug delivery across mucosal barriers. Acta Biomater. 119, 13–29. 10.1016/j.actbio.2020.10.031 33141051

[B5] Bayer I. S. (2022). Recent advances in mucoadhesive interface materials, mucoadhesion characterization, and technologies. Adv. Mater. Interfaces. 9, 2200211. 10.1002/admi.202200211

[B6] Buchsel P. C. (2008). Polyvinylpyrrolidone-sodium hyaluronate gel (Gelclair): A bioadherent oral gel for the treatment of oral mucositis and other painful oral lesions. Expert Opin. Drug Metab. Toxicol. 4 (11), 1449–1454. 10.1517/17425255.4.11.1449 18950285

[B7] Caramella C. M. Bonferoni M. C. Sandri G. Dellera E. Rossi S. Ferrari F. (2015). “Medical devices for oral mucosal applications,” in Oral mucosal drug delivery and therapy. Editors Rathbone M. J. Senel S. Pather I. (Springer), 225–245. Chapter 10, ISBN: 978-1-4899-7557-7. (a).

[B8] Caramella C. M. Rossi S. Ferrari F. Bonferoni M. C. Sandri G. (2015b). Mucoadhesive and thermogelling systems for vaginal drug delivery. Adv. Drug Deliv. Rev. 92, 39–52. 10.1016/j.addr.2015.02.001 25683694

[B9] Carvalho F. C. Bruschi M. L. Evangelista R. C. Gremião M. P. D. (2010). Mucoadhesive drug delivery systems. Br. J. Pharm. Sci. 46, 1–17. 10.1590/S1984-82502010000100002

[B10] Carvalho F. C. Calixto G. Hatakeyama I. N. Marielli Luz G. Daflon Gremião M. P. Chorilli M. (2013). Rheological, mechanical, and bioadhesive behavior of hydrogels to optimize skin delivery systems. Drug Dev. Ind. Pharm. 39 (11), 1750–1757. 10.3109/03639045.2012.734510 23216218

[B11] Chatterjee B. Amalina N. Sengupta P. Mandal U. K. (2017). Mucoadhesive polymers and their mode of action: A recent update. J. App. Pharm. Sci. 7, 195–203. 10.7324/JAPS.2017.70533

[B12] Collado-González M. González Espinosa Y. Goycoolea F. M. (2019). Interaction between chitosan and mucin: Fundamentals and applications. Biomimetics 4 (2), 32. 10.3390/biomimetics4020032 31105217 PMC6631199

[B13] Cook M. T. Khutoryanskiy V. V. (2015). Mucoadhesion and mucosa-mimetic materials--A mini-review. Int. J. Pharm. 495, 991–998. 10.1016/j.ijpharm.2015.09.064 26440734

[B14] da Silva J. B. Dos Santos R. S. Vecchi C. F. Bruschi M. L. (2022). Drug delivery platforms containing thermoresponsive polymers and mucoadhesive cellulose derivatives: A review of patents. Recent Adv. Drug Deliv. Formul. 16 (2), 90–102. 10.2174/2667387816666220404123625 35379163

[B15] Dalei G. Das S. (2022). Polyacrylic acid-based drug delivery systems: A comprehensive review on the state-of-art. J. Drug Deliv. Sci. Technol. 78, 103988. 10.1016/j.jddst.2022.103988

[B16] de Lima C. S. A. Varca J. P. R. O. Alves V. M. Nogueira K. M. Cruz C. P. C. Rial-Hermida M. I. (2022). Mucoadhesive polymers and their applications in drug delivery systems for the treatment of bladder cancer. Gels 8 (9), 587. 10.3390/gels8090587 36135300 PMC9498303

[B17] Di Simone M. P. Baldi F. Vasina V. Scorrano F. Bacci M. L. Ferrieri A. (2012). Barrier effect of Esoxx(®) on esophageal mucosal damage: Experimental study on *ex-vivo* swine model. Clin. Exp. Gastroenterol. 5, 103–107. 10.2147/CEG.S31404 22767997 PMC3387832

[B18] Dolci L. S. Albertini B. Di Filippo. M. F. Bonvicini F. Passerini N. Panzavolta S. (2020). Development and *in vitro* evaluation of mucoadhesive gelatin films for the vaginal delivery of econazole. Int. J. Pharm. 591, 119979. 10.1016/j.ijpharm.2020.119979 33068694

[B19] Dovedytis M. Liu Z. J. Bartlett S. (2020). Hyaluronic acid and its biomedical applications: A review. E. R. 1, 102–113. 10.1016/j.engreg.2020.10.001

[B20] Duchene D. Touchard F. Peppas N. A. (1988). Pharmaceutical and medical aspects of bioadhesive systems for drug administration. Drug Dev. Ind. Pharm. 14, 283–318. 10.3109/03639048809151972

[B21] Duggan S. Cummins W. O' Donovan O. Hughes H. Owens E. (2017). Thiolated polymers as mucoadhesive drug delivery systems. Eur. J. Pharm. Sci. 100, 64–78. 10.1016/j.ejps.2017.01.008 28087353

[B22] Edsman K. Hägerström H. (2005). Pharmaceutical applications of mucoadhesion for the non-oral routes. J. Pharm. Pharmacol. 57 (1), 3–22. 10.1211/0022357055227 15638988

[B23] Estrellas K. M. Fiecas M. Azagury A. Laulicht B. Cho D. Y. Mancini A. (2019). Time-dependent mucoadhesion of conjugated bioadhesive polymers. Colloids Surf. B Biointerfaces. 173, 454–469. 10.1016/j.colsurfb.2018.10.011 30326362

[B24] European Commission (2009). DG enterprise and industry. Medical devices: Guidance document: Borderline products, drug-delivery products and medical devices incorporating, as an integral part, an ancillary medicinal substance or an ancillary human blood derivative. MEDDEV 2.1/3 rev. 3 2009. Guidelines relating to the application of:the Council Directive 90/385/EEC on active implantable medical devices and the Council Directive 93/42/EEC on medical devices.

[B25] European Union (1993). Council Directive 93/42/EEC of 14 June 1993 concerning medical devices. Official Journal of the European Communities no. L-169 of 12 July 1993.

[B26] European Union (2017). Regulation (EU) 2017/745 of the European parliament and of the council of 5 April 2017 on medical devices, amending directive 2001/83/EC, regulation (EC) No 178/2002 and regulation (EC) No 1223/2009 and repealing council directives 90/385/EEC and 93/42/EEC.

[B27] Fallacara A. Baldini E. Manfredini S. Vertuani S. (2018). Hyaluronic acid in the third millennium. Polym. (Basel) 10 (7), 701. 10.3390/polym10070701 PMC6403654 30960626

[B28] Federer C. Kurpiers M. Bernkop-Schnürch A. (2021). Thiolated chitosans: A multi-talented class of polymers for various applications. Biomacromolecules 22 (1), 24–56. 10.1021/acs.biomac.0c00663 32567846 PMC7805012

[B29] Freitas E. D. Moura C. F. Kerwald J. Beppu M. M. (2020). An overview of current knowledge on the properties, synthesis and applications of quaternary chitosan derivatives. Polymers 12 (12), 2878. 10.3390/polym12122878 33266285 PMC7759937

[B30] Göbel A. Bassi da Silva J. Cook M. Breitkreutz J. (2021). Development of buccal film formulations and their mucoadhesive performance in biomimetic models. Int. J. Pharm. 610, 121233. 10.1016/j.ijpharm.2021.121233 34710543

[B31] Gosecka M. Gosecki M. (2021). Antimicrobial polymer-based hydrogels for the IntravaginalTherapies-engineering considerations. Pharmaceutics 13, 1393. 10.3390/pharmaceutics13091393 34575468 PMC8469626

[B32] Gu J. M. Robinson J. R. Leung S. H. S. (1988). Binding of acrylic polymers to mucin/epithelial surfaces: Structure property relationships. Crit. Rev. Ther. Drug Carr. Sys. 5, 21–67. 3293807

[B33] Hägerström H. Edsman K. Strømme M. (2003). Low-frequency dielectric spectroscopy as a tool for studying the compatibility between pharmaceutical gels and mucous tissue. J. Pharm. Sci. 92 (9), 1869–1881. 10.1002/jps.10451 12950005

[B34] Hamza O. J. M. Matee M. I. N. Brüggemann R. J. M. Moshi M. J. Simon E. N. M. Mugusi F. (2008). Single-dose fluconazole versus standard 2-week therapy for oropharyngeal candidiasis in HIV-infected patients: A randomized, double-blind, double-dummy trial. Clin. Infect. Dis. 47 (10), 1270–1276. 10.1086/592578 18840077

[B35] Hanafy N. A. N. Leporatti S. El-Kemary M. A. (2019). Mucoadhesive hydrogel nanoparticles as Smart biomedical drug delivery system. App. Sci. 9, 825. 10.3390/app9050825

[B36] Huang Y. Leobandung W. Foss A. Peppas N. A. (2000). Molecular aspects of muco- and bioadhesion: Tethered structures and site-specific surfaces. J. Control. Release. 65 (1-2), 63–71. 10.1016/s0168-3659(99)00233-3 10699271

[B37] Jiménez M. M. Fresno M. J. Ramírez A. (2007). Rheological study of binary gels with Carbopol Ultrez 10 and hyaluronic acid. Chem. Pharm. Bull. 55 (8), 1157–1163. 10.1248/cpb.55.1157 17666837

[B38] Jimenez-Castellanos M. R. Zia H. Rhodes C. T. (1993). Mucoadhesive drug delivery systems. Drug Dev. Ind. Pharm. 19, 143–194. 10.3109/03639049309038765

[B39] Karavasili C. Eleftheriadis G. K. Gioumouxouzis C. Andriotis E. G. Fatouros D. G. (2021). Mucosal drug delivery and 3D printing technologies: A focus on special patient populations. Adv. Drug Deliv. Rev. 176, 113858. 10.1016/j.addr.2021.113858 34237405

[B40] Khutoryanskiy V. V. (2011). Advances in mucoadhesion and mucoadhesive polymers. Macromol. Biosci. 11 (6), 748–764. 10.1002/mabi.201000388 21188688

[B41] Knoll P. Le N. N. Wibel R. Baus R. A. Kali G. Asim M. H. (2021). Thiolated pectins: *In vitro* and *ex vivo* evaluation of three generations of thiomers. Acta Biomater. 135, 139–149. 10.1016/j.actbio.2021.08.016 34418540

[B42] Kumar A. Kumar Naik P. Pradhan D. Ghosh G. Rath G. (2020). Mucoadhesive formulations: Innovations, merits, drawbacks, and future outlook. Pharm. Dev. Technol. 25 (7), 797–814. 10.1080/10837450.2020.1753771 32267180

[B43] Kumar A. Vimal A. Kumar A. (2016). Why chitosan? From properties to perspective of mucosal drug delivery. Int. J. Biol. Macromol. 91, 615–622. 10.1016/j.ijbiomac.2016.05.054 27196368

[B44] Lam H. T. Zupancic O. Laffleur F. Bernkop-Schnurch A. (2021). Mucoadhesive properties of polyacrylates: Structure – function relationship. Int. J. Adhes. Adhes. 107, 102857. 10.1016/j.ijadhadh.2021.102857

[B45] Lee J. W. Park J. H. Robinson J. R. (2000). Bioadhesive-based dosage forms: The next generation. J. Pharm. Sci. 89 (7), 850–866. 10.1002/1520-6017(200007)89:7<850::AID-JPS2>3.0.CO;2-G 10861586

[B46] Leichner C. Jelkmann M. Bernkop-Schnürch A. (2019). Thiolated polymers: Bioinspired polymers utilizing one of the most important bridging structures in nature. Adv. Drug Deliv. Rev. 151-152, 191–221. 10.1016/j.addr.2019.04.007 31028759

[B47] Leitner V. M. Marschütz M. K. Bernkop-Schnürch A. (2003). Mucoadhesive and cohesive properties of poly(acrylic acid)-cysteine conjugates with regard to their molecular mass. Eur. J. Pharm. Sci. 18 (1), 89–96. 10.1016/s0928-0987(02)00245-2 12554077

[B48] Marcilla A. Beltran M. (2004). Mechanisms of plasticizers action. Handb. plasticizers 2004, 107–120.

[B49] Mathiowitz E. Chickering D. E. Lehr C. M. (Editors) (1999). “Bioadhesive drug delivery systems: Fundamentals, novel approaches, and development,” Drugs and the pharmaceutical sciences (New York: Marcel Dekker), 696.

[B50] Mayol L. Quaglia F. Borzacchiello A. Ambrosio L. La Rotonda M. I. (2008). A novel poloxamers/hyaluronic acid *in situ* forming hydrogel for drug delivery: Rheological, mucoadhesive and *in vitro* release properties. Eur. J. Pharm. Biopharm. 70 (1), 199–206. 10.1016/j.ejpb.2008.04.025 18644705

[B51] McBain J. W. Hopkins D. G. (2002). On adhesives and adhesive action. J. Phys. Chem. 29 (2), 188–204. 10.1021/j150248a008

[B52] Mura P. Maestrelli F. Cirri M. Mennini N. (2022). Multiple roles of chitosan in mucosal drug delivery: An updated review. Mar. Drugs. 20 (5), 335. 10.3390/md20050335 35621986 PMC9146108

[B53] Park H. Robinson J. R. (1987). Mechanisms of Mucoadhesion of Poly(acrylic acid) hydrogels. Pharm. Res. 4 (6), 457–464. 10.1023/a:1016467219657 3508557

[B54] Patel M. M. Smart J. D. Nevell T. G. Ewen R. J. Eaton P. J. Tsibouklis J. (2003). Mucin/poly(acrylic acid) interactions: A spectroscopic investigation of mucoadhesion. Biomacromolecules 4 (5), 1184–1190. 10.1021/bm034028p 12959582

[B55] Peppas N. A. Buri P. A. (1985). Surface, interfacial and molecular aspects of polymer bioadhesion on soft tissues. J. Control Release 2, 257–275. 10.1016/0168-3659(85)90050-1

[B56] Peppas N. A. Sahlin J. J. (1996). Hydrogels as mucoadhesive and bioadhesive materials: A review. Biomaterials 17 (16), 1553–1561. 10.1016/0142-9612(95)00307-x 8842358

[B57] Pritchard K. Lansley A. B. Martin G. P. Helliwell M. Marriott C. Benedetti C. M. (1996). Evaluation of the bioadhesive properties of hyaluronan derivatives: Detachment weight and mucociliary transport rate studies. Int. J. Pharm. 129, 137–145. 10.1016/0378-5173(95)04280-6

[B58] Racchi M. Govoni S. Lucchelli A. Capone L. Giovagnoni E. (2016). Insights into the definition of terms in European medical device regulation. Expert Rev. Med. devices. 13 (10), 907–917. 10.1080/17434440.2016.1224644 27559622

[B59] Reinhart C. T. Peppas N. A. (1984). Solute diffusion in swollen membranes. Part II. Influence of crosslinking on diffusive properties. J. Memb. Sci. 18, 227–239. 10.1016/S0376-7388(00)85036-X

[B60] Rossi S. Bonferoni M. C. D’Autilia F. Sandri G. Ferrari F. Caramella C. (2014). Associations of natural polymers to modulate mucoadhesion of vaginal rinse-off and leave-on formulations. J. Drug Deliv. Sci. Tec. 24 (5), 496–502. 10.1016/s1773-2247(14)50094-9

[B61] Rossi S. Bonferoni M. C. Ferrari F. Caramella C. (1999). Drug release and washability of mucoadhesive gels based on sodium carboxymethylcellulose and polyacrylic acid. Pharm. Dev. Technol. 4 (1), 55–63. 10.1080/10837459908984224 10027213

[B62] Rossi S. Vigani B. Bonferoni M. C. Sandri G. Caramella C. Ferrari F. (2018). Rheological analysis and mucoadhesion: A 30 year-old and still active combination. J. Pharm. Biomed. Anal. 156, 232–238. 10.1016/j.jpba.2018.04.041 29729636

[B63] Roy S. Pal K. Anis A. Pramanik K. Prabhakar B. (2009). Polymers in mucoadhesive drug-delivery systems: A brief note. Monomers Polym. 12, 483–495. 10.1163/138577209X12478283327236

[B64] Salamat-Miller N. Chittchang M. Johnston T. P. (2005). The use of mucoadhesive polymers in buccal drug delivery. Adv. Drug Deliv. Rev. 57 (11), 1666–1691. 10.1016/j.addr.2005.07.003 16183164

[B65] Sandri G. Bonferoni M. C. Rossi S. Ferrari F. Gibin S. Zambito Y. (2007). Nanoparticles based on N-trimethylchitosan: Evaluation of absorption properties using *in vitro* (Caco-2 cells) and *ex vivo* (excised rat jejunum) models. Eur. J. Pharm. Biopharm. 65 (1), 68–77. 10.1016/j.ejpb.2006.07.016 16962751

[B66] Sandri G. Rossi S. Bonferoni M. C. Ferrari F. Mori M. Caramella C. (2012). The role of chitosan as a mucoadhesive agent in mucosal drug delivery. J. Drug Deliv. Sci. Technol. 22, 275–284. 10.1016/S1773-2247(12)50046-8

[B67] Sandri G. Rossi S. Ferrari F. Bonferoni M. C. Caramella C. (2015). “Mucoadhesive polymers as enabling excipients for oral mucosal drug delivery,” in Oral mucosal drug delivery and therapy. Editors Rathbone M. J. Senel S. Pather I. (Springer). Chapter 4, 53-88, ISBN: 978-1-4899-7557-7.

[B68] Sandri G. Rossi S. Ferrari F. Bonferoni M. C. Zerrouk N. Caramella C. (2004). Mucoadhesive and penetration enhancement properties of three grades of hyaluronic acid using porcine buccal and vaginal tissue, Caco-2 cell lines, and rat jejunum. J. Pharm. Pharmacol. 56 (9), 1083–1090. 10.1211/0022357044085 15324476

[B69] Shaikh R. Singh T. R. R. Garland M. J. Woolfson A. D. Donnelly R. F. (2011). Mucoadhesive drug delivery systems. J. Pharm. Bioallied Sci. 3 (1), 89–100. 10.4103/0975-7406.76478 21430958 PMC3053525

[B70] Shinkar D. M. Dhake A. S. Setty C. M. (2012). Drug delivery from the oral cavity: A focus on mucoadhesive buccal drug delivery systems. PDA J. Pharm. Sci. Technol. 66, 466–500. 10.5731/pdajpst.2012.00877 23035030

[B71] Smart J. D. (2005). The basics and underlying mechanisms of mucoadhesion. Adv. Drug Deliv. Rev. 57 (11), 1556–1568. 10.1016/j.addr.2005.07.001 16198441

[B72] Sosnika A. das Nevesb J. Sarmentob B. (2014). Mucoadhesive polymers in the design of nano-drug deliverysystems for administration by non-parenteral routes: A review. Prog. Polym. Sci. 39, 2030–2075. 10.1016/j.progpolymsci.2014.07.010

[B73] Suharyani I. Mohammed A. F. A. Muchtaridi M. Wathoni N. Abdassah M. (2021). Evolution of drug delivery systems for recurrent aphthous stomatitis. Drug Des. devel. Ther. 15, 4071–4089. 10.2147/DDDT.S328371 PMC8489189 34616142

[B74] Summonte S. Racaniello G. F. Lopedota A. Denora N. Bernkop-Schnürch A. (2021). Thiolated polymeric hydrogels for biomedical application: Cross-linking mechanisms. J. Control. Release 330, 470–482. 10.1016/j.jconrel.2020.12.037 33359581

[B75] Surendranath M. Rekha M. R. Parameswaran R. (2022). Recent advances in functionally modified polymers for mucoadhesive drug delivery. J. Mater. Chem. B 10 (31), 5913–5924. 10.1039/d2tb00856d 35880449

[B76] Tamburic S. Craig D. Q. M. (1995). An investigation into the rheological, dielectric and mucoadhesive properties of poly(acrylic acid) gel systems. J. Control. Release. 37 (1-2), 59–68. 10.1016/0168-3659(95)00064-F 8932449

[B77] Vasvani S. Kulkarni P. Rawtani D. (2020). Hyaluronic acid: A review on its biology, aspects of drug delivery, route of administrations and a special emphasis on its approved marketed products and recent clinical studies. Int. J. Biol. Macromol. 151, 1012–1029. 10.1016/j.ijbiomac.2019.11.066 31715233

[B78] Vigani B. Rossi S. Sandri G. Bonferoni M. C. Caramella C. M. Ferrari F. (2019). Hyaluronic acid and chitosan-based nanosystems: A new dressing generation for wound care. Expert Opin. Drug Deliv. 16 (7), 715–740. 10.1080/17425247.2019.1634051 31215823

[B79] Vokurka S. Skardova J. Hruskova R. Kabatova-Maxova K. Svoboda T. Bystricka E. (2011). The effect of polyvinylpyrrolidone-sodium hyaluronate gel (Gelclair) on oral microbial colonization and pain control compared with other rinsing solutions in patients with oral mucositis after allogeneic stem cells transplantation. Med. Sci. Monit. 17 (10), CR572–6. 10.12659/msm.881983 21959611 PMC3539478

[B80] Wahlgren M. Löwenstein C. K. Valentin Jørgensen E. Svensson A. Ulvenlund S. (2009). Oral-based controlled release formulations using poly(acrylic acid) microgels. Drug Dev. Ind. Pharm. 35 (8), 922–929. 10.1080/03639040802698810 19466881

[B81] Ways T. M. M. Lau W. M. Khutoryanskiy V. V. (2018). Chitosan and its derivatives for application in mucoadhesive drug delivery systems. Polymers 10 (3), 267. 10.3390/polym10030267 30966302 PMC6414903

[B82] Yermak I. M. Davydova V. N. Volod'ko A. V. (2022). Mucoadhesive marine polysaccharides. Mar. Drugs. 20 (8), 522. 10.3390/md20080522 36005525 PMC9409912

[B83] Zahir-Jouzdani F. Wolf J. D. Atyabi F. Bernkop-Schnürch A. (2018). *In situ* gelling and mucoadhesive polymers: Why do they need each other? Expert opin. Drug Deliv. 15 (10), 1007–1019. 10.1080/17425247.2018.1517741 30173567

